# A Case Report on CNS Hemophagocytic Lymphohistiocytosis in an Infant With Dengue Hemorrhagic Fever

**DOI:** 10.7759/cureus.34773

**Published:** 2023-02-08

**Authors:** Amodini Arora, Sarita Verma, Nikita Khot, Shiji Chalipat, Sharad Agarkhedkar, Kala Gnanasekaran Kiruthiga

**Affiliations:** 1 Pediatrics, Dr. D.Y. Patil Medical College, Pune, IND; 2 Pediatric Oncology, KEM Hospital & Research Centre, Pune, IND; 3 Pediatric Oncology, Dr. D.Y. Patil Medical College, Pune, IND; 4 Histopathology, KEM Hospital & Research Centre, Pune, IND

**Keywords:** cns involvement, dengue with warning signs, dengue fever/complications, cns manifestations, pediatric infectious disease, neurology, paediatrics, hemophagocytic lymphohistiocytosis (hlh), dengue hemorrhagic fever (dhf)

## Abstract

India is an endemic country for dengue. The incidence of hemophagocytic lymphohistiocytosis (HLH) with dengue in children has been well-reported. However, central nervous system (CNS) HLH associated with dengue has not been described in the literature yet. We hereby report a novel case of CNS HLH triggered by dengue infection. An eight-month-old, well-grown male infant with uneventful antenatal, perinatal, and neonatal history was admitted with a history of febrile illness associated with cough, cold, vomiting, and loose motions and one episode of hematochezia and hepatosplenomegaly on examination. Investigations revealed bi-cytopenia, hyper-ferritinemia, deranged coagulation profile, liver function test, and hypo-fibrinogenemia. Dengue non-structural protein 1 ( NS1) antigen was positive. The child was given dexamethasone and continued supportive care with a diagnosis of dengue shock syndrome. The child showed an overall transient improvement, however, he had rebound fever followed by right focal convulsion on Day 9 of steroids. MRI brain revealed areas of diffusion-restricted embolic infarcts with diffuse leptomeningeal enhancement and mild cerebral edema, and CSF showed a total leukocyte count of 80 cells with 75% lymphocytic picture, histiocytes with hemophagocytosis, confirmatory of CNS HLH. Intrathecal methotrexate, hydrocortisone, and intravenous (IV) etoposide were started. However, the child succumbed to his illness. CNS involvement in dengue-triggered HLH needs to be suspected despite subtle neurological signs and aggressively managed following a multi-departmental approach to ensure the best clinical and neuro-developmental outcomes.

## Introduction

The incidence of hemophagocytic lymphohistiocytosis (HLH) with dengue in children has been well-reported [1]. India contributed 34 of 96 million apparent global dengue infections, a number which stands in stark contrast to the 12,484 reported cases from India to the WHO in the same year [2]. Recently, dengue has been associated with hyper-inflammation and hyper-ferritinemia with or without HLH, an uncommon fatal syndrome. It is called primary HLH when caused by genetic mutations responsible for the production of T cells and natural killer (NK) cells. It can be secondary when triggered by malignant or non-malignant diseases like juvenile idiopathic arthritis (JIA), systemic lupus erythematosus (SLE), other autoimmune diseases, and infections like Epstein Barr Virus (EBV), human immunodeficiency virus (HIV), cytomegalovirus (CMV), tuberculosis (TB), dengue and possibly severe acute respiratory syndrome coronavirus 2 (SARS-CoV-2) [3]. Timely administration of steroids has led to improved outcomes in HLH with dengue [4]. The incidence of HLH with dengue in children has been well-reported [4]. However, CNS HLH associated with dengue has not been described in the literature yet. We hereby report a novel case of CNS HLH in an infant triggered by dengue infection.

## Case presentation

An eight-month-old, well-grown male infant with an uneventful antenatal, perinatal, and neonatal course was admitted with febrile illness associated with cough, cold, vomiting, and loose motions for 10 days and one episode of hematochezia a day before admission. Clinical examination revealed tachycardia (HR 174), hypotension (78/32 mmHg - at the 5th percentile), and delayed capillary refill time (4 seconds). On abdominal examination, there was hepato-splenomegaly. Neurologically, the child’s consciousness was obtunded (the Alert/Voice/Pain/Unresponsive (AVPU) scale - Pain).

The child was admitted to the pediatric intensive care Unit. After initial stabilization, fluid resuscitation and supportive treatment were started. Several crucial investigations were done, including a hemogram, which revealed a bi-cytopenic picture (Table 1). Liver function tests were grossly deranged with serum glutamic oxaloacetic transaminase - 18,261 IU/L, serum glutamic pyruvic transaminase - 7834 IU/L, and a deranged coagulation profile with international normalized ratio (INR) 4.11 was noted. Ferritin was more than 40,000 ng/ml, lactate dehydrogenase (LDH) was 9116 IU/L, fibrinogen was 75.6 mg/dl, and triglycerides were 278 mg/dl; most of these reports were strongly suggestive of HLH. He was dengue non-structural protein 1 (NS1) antigen-positive. Immunoglobulin M (IgM) and IgG were negative with a normal chest X-ray and 2-dimensional echocardiography. IV dexamethasone was started at 10 mg/m^2^ on the first day of admission itself, based on clinical and laboratory confirmation of dengue-triggered HLH.

**Table 1:** Comparison of the serial investigations of the patient

DAY	Day 1	Day 2	Day 3	Day 4	Day 5	Day 6	Day 7	Day 8	Day 9	Day 10	Day 11	Day 12
Haemoglobin (g/dl) (Reference Range - 11.14-14.1g/dl)	7.7	11.2	9.8	8.8	9.8	12	8.5	7.9	5.4	11.8	10.8	9.5
Total Leukocyte Count ( per microliter) (Reference Range- 6000-11,000/microliter)	20500	14300	8600	9300	8700	13300	12600	11100	7100	9300	8200	3400
Differential leukocyte count (Neutrophil/Lymphocyte/Eosinophil) (%) Reference Range - Neutrophil (1500-8500/microliter), Lymphocytes (4000-10,000 /microliter), Eosinophils (50-700/microliter)	46/34/20	51/34	46/41	47/40	56/29	76/15	77/15/8	79/15	66/25	76/16	79/18	70/30
Platelets (per microliter) (Reference Range- 1,50,000-4,10,000/microliter)	44000	78000	61000	62000	82000	42000	90000	78000	32000	49000	40000	14000
Packed cell volume (%) (Reference Range -33.0-40.0%)	22.4	33.4	28.1	26	29.3	36.1	27.1	24.7	17	36.2	33.4	29.4
Aspartate transaminase (Units/Liter) (Reference Range 0-11 months-Not established, 1-13 years - 8 to 60 U/liter)	18261	8404	7132	3134	2067	1374	549		231	186		183
Alanine transaminase (Units/Liter) (Reference Range- 0 to 11 months-Not Established, 1-13 years - 8 to 60 U/Liter)	7834	4113	4266	2680	2294	1863	1236		698	625		512
Lactate dehydrogenase (Units/Liter) (Reference Range - 31 days to 11 months -180-435 U/Liter)	9116	4921										
Ferritin (Nanogram/ml) (Reference Range - 21.81 to 274.66 ng/ml)	>40000	37964	23169		8125				3324			

As shown in Table 1, a repeat hemogram post 24 hours showed an improvement in the platelet count and general condition of the child. The child became afebrile after 48 hours of dexamethasone and the blood indices improved with rising platelet count and total leukocytes. Hence, steroids with other supportive treatments were continued. The serum ferritin improved to 8125 ng/ml on Day 6 of dexamethasone.

The child was shifted out of the pediatric intensive care unit (PICU) after seven days of intensive support. On Day 9 of dexamethasone, the child had a rebound fever spike followed by right-sided focal convulsions and a right-sided focal neurological deficit with hemiparesis, facial nerve palsy, and altered sensorium. After initial stabilization, neuroimaging followed by a lumbar puncture was done.

**Figure 1:**
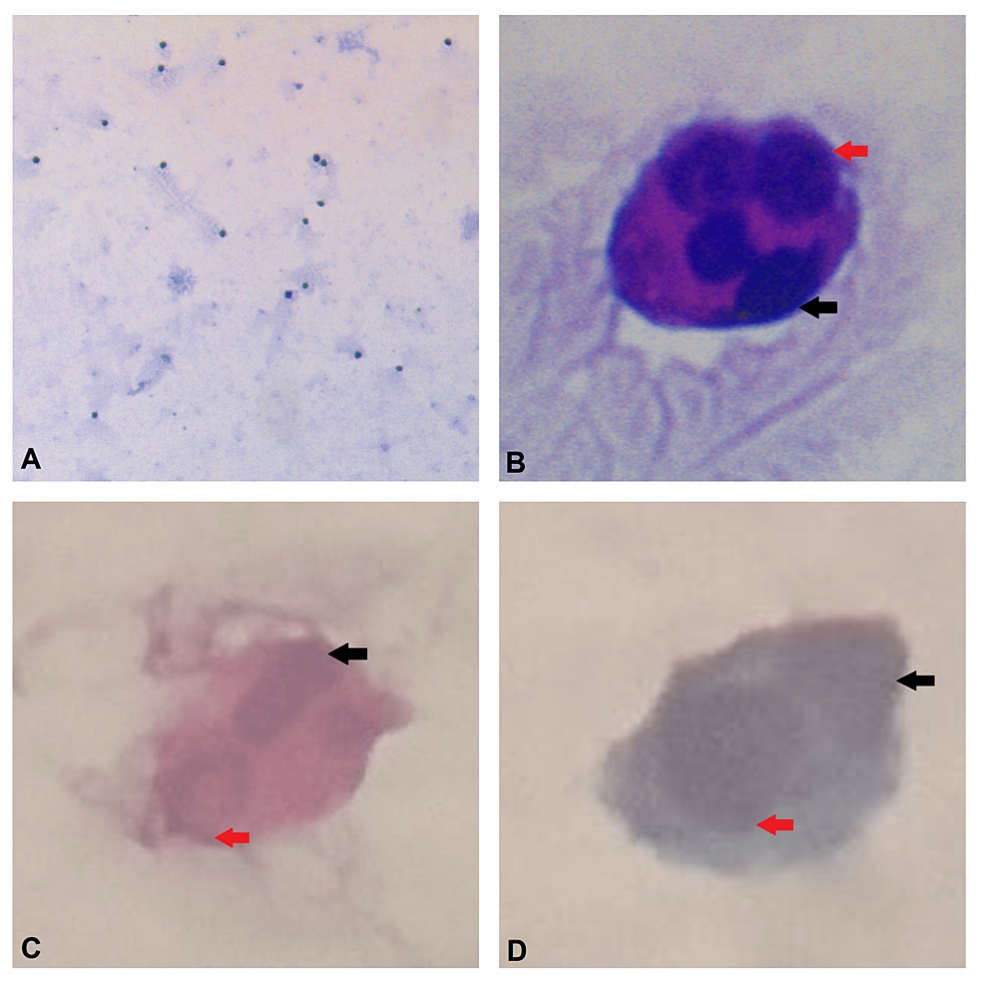
Cerebrospinal fluid cytology The black arrow represents the nucleus of the macrophage/histiocyte. A. Pleocytosis of CSF; B. Macrophage engulfing a neutrophil (red arrow), Leishman stain; C. Macrophage engulfing a lymphocyte (red arrow), hematoxylin and eosin stain; D. Macrophage engulfing a lymphocyte (red arrow), Papanicolaou stain

**Figure 2:**
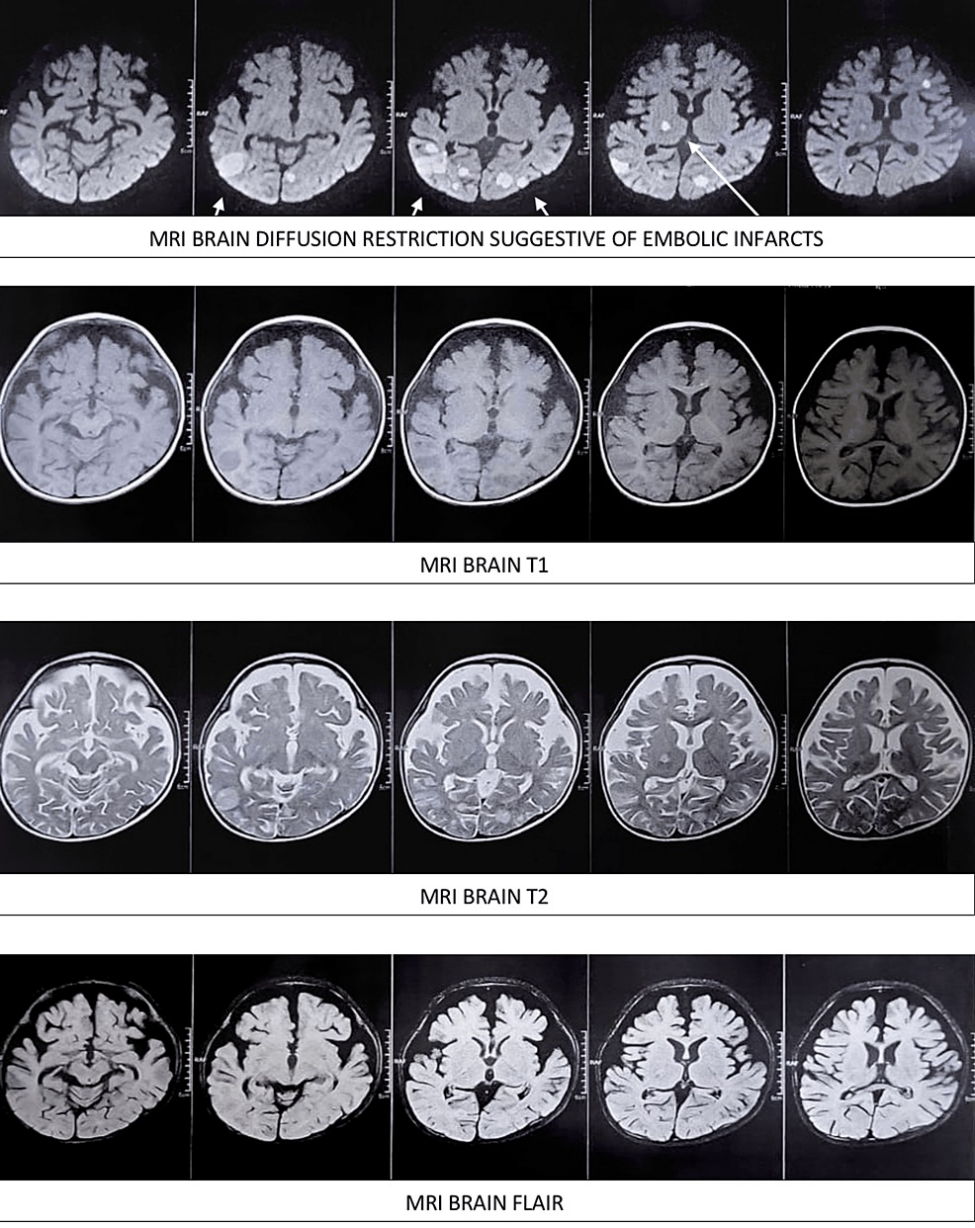
Magnetic resonance imaging (MRI) brain (diffusion restriction, T1, T2, Flair) (top to bottom) revealed areas of diffusion-restricted embolic infarcts with diffuse leptomeningeal enhancement and mild cerebral edema

Cerebrospinal fluid examination showed histiocytes with hemophagocytosis confirming hemophagocytic histiocytosis (CNS HLH) along with absent cob-webs, proteins - 61.70 mg/dl, glucose - 64 mg/dl, red blood cells - absent, total leukocyte count - 80 cells, polymorphonuclear leukocytes - 5% and lymphocytes - 75%, macrophages - 20%, pleomorphic cells - 0, adenosine deaminase (ADA) - 8.21 IU/L (Figure 1). Magnetic resonance imaging (MRI) brain revealed areas of diffusion-restricted embolic infarcts with diffuse leptomeningeal enhancement and mild cerebral edema (Figure 2).

The child was given intrathecal methotrexate and hydrocortisone with IV etoposide as per the Hemophagocytic Lymphohistiocytosis 2004 protocol [5], however, the patient remained persistently febrile and the pancytopenia worsened despite granulocyte colony-stimulating factor (G-CSF) support. The child also deteriorated hemodynamically and neurologically and required mechanical ventilation. Despite all resuscitative measures, the child succumbed to his illness 72 hours post receiving intrathecal chemotherapy.

## Discussion

Viral infections commonly trigger pathologies like HLH; however, the exact mechanisms of viral implication in the pathogenesis of HLH are unproven. Some studies postulate viruses, as potent modulators of the immune system, causing evasion of interference with the cytokine balance, immune recognition, and hindrance of apoptotic pathways. Some theories suggest abnormally proliferating as well as activated T-cells as possible causes of activation of macrophages and sub-normal destruction of phagocytes. Others postulated the role of perforin and natural killer (NK) cells. A deficiency of perforin can impair cellular defenses and reduced NK cell activity causes greater T-cell functioning and significant amounts of cytokine production (IFN-γ, TNF-α, GM-CSF), causing persistent macrophage activation. These findings are commonly triggered by viral pathologies. Even though macrophages phagocytize infected leukocytes, they are not very effective in killing them. Active T cells along with plenty of activated macrophages significantly increase inflammatory cytokine production resulting in greater damage to bone marrow and tissues. The invariant natural killer T (iNKT) cells are also activated during acute phases of dengue; they are poly-functional and produce IFN-γ and GM-CSF on stimulation. Detailed studies are required to clarify the pathogenesis of HLH and dengue.

The most recent criteria for diagnosis of HLH include fever, splenomegaly, cytopenias, hypertriglyceridemia and/or hypofibrinogenemia, hemophagocytosis, decreased natural killer (NK) cell function, and elevated ferritin and elevated soluble interleukin-2 (IL-2) receptor levels. CNS involvement was not included in the criteria and can include seizures, focal deficits, meningismus, and altered levels of consciousness; and portends worse outcomes [6]. Severe dengue infection complicated with HLH requires very prompt and aggressive treatment with steroids or intravenous immune globulin or chemotherapy [7]. Many reports of secondary HLH triggered by dengue infection have been reported [6-8]; however, none of them had CNS HLH documented. We, for the first time, report on confirmed CNS HLH triggered by dengue infection in an eight-month-old infant. Our patient had all the laboratory markers confirmatory to HLH at presentation as per HLH 2004 protocol; hence, was immediately treated with steroids within 24 hours. The child showed significant transient responses like improvement in Glasgow Coma Scale decreasing inflammatory markers and rising blood indices; however, the acute unpredictable neurological worsening in this infant emphasizes the need of suspecting CNS involvement in dengue-triggered secondary HLH because it warrants specific intrathecal chemotherapy. Genetic evaluation could not be performed, hence the possibility of congenital HLH being precipitated with dengue infection always remains. Though primary HLH always remains a differential, the treatment in the acute scenario is the same. Whether CSF examination in all patients with HLH is warranted and needs to be addressed with subsequent studies on a larger population.

## Conclusions

Hemophagocytic lymphohistiocytosis has proven to be a potentially lethal pathology with multisystemic manifestations, especially in developing countries like India. However, CNS involvement secondary to HLH is an infrequent finding. To differentiate HLH from the underlying disease mechanism or overt septicemia is imperative and taxing owing to high mortality and morbidity. Steroids alone are insufficient for treating CNS HLH. Dengue hemorrhagic fever has been a strongly and commonly documented trigger, A diagnosis of HLH should be considered in every sick child presenting with bi-cytopenia and hyperferritinemia.

If there is CNS HLH in the form of aseptic meningitis or the frank morphological presence of macrophages and hemophagocytes in the CSF, aggressive treatment with intrathecal methotrexate and intrathecal steroids should be initiated immediately. The addition of systemic etoposide with cyclosporine in patients not improving despite steroids can prove to be potentially life-saving. A take-home message on the basis of a single novel experience is the importance of CSF screening in relevant scenarios (subtle neurological symptoms) for the timely diagnosis and treatment of CNS involvement in dengue-triggered HLH. We highlight the importance of a multi-departmental approach with pediatric intensivists working in close proximity with pediatric neurologists, hemato-oncologists, histopathologists, and microbiologists to devise a comprehensive plan of action with a focus on the best clinical and neuro-developmental outcomes.
